# The *new why* when designing mandatory medical examinations

**DOI:** 10.1093/occmed/kqx044

**Published:** 2017-07-15

**Authors:** Judith K. Sluiter

**Affiliations:** 1 Coronel Institute of Occupational Health, Academic Medical Center, University of Amsterdam,, Amsterdam Public Health Research Institute, , PO Box 22700, 1100 DE Amsterdam, The Netherlands

The basic medical training of physicians is almost completely carried out in hospitals using a traditional medical model and this may be less relevant for future preventive work in public or occupational medicine. This is especially the case when performing mandatory medical examinations on workers where the traditional approach hampers good practice. The ‘*why*’, when thinking about mandatory medical examinations in workers, could be described as to periodically verify, given some specific and special working conditions, whether an employee can perform his or her job safely without an increased health risk [1]. National laws, regulations and professional practice guidelines are responsible for differences between countries in how occupational medicine professionals deal with the content (‘*the what*’) and procedures (‘*the how*’) around mandatory medical examinations of workers. The different terminology that is used seems to be the least of the problems: pre-placement health assessments, pre-employment medicals, on-employment medical examinations, fit-to-work medical assessments, etc., because they all speak more or less for themselves. Sectoral differences are obvious because for jobs in some sectors the boundaries between countries are less-existent and therefore applying the same criteria seems logical when a worker crosses countries on a daily basis (i.e. seafarers, train drivers, pilots, professional divers or jobs in the off-shore industry). For these jobs, international consensus guidelines among medical professionals on the content of mandatory examinations have been around for decades. Unfortunately, the classical clinical way of thinking (looking for symptoms, signs and diseases) was predominantly used when designing ‘*the what*’ in these medical examinations: long lists of diseases had to be checked off by the physician and questions about health history were normal. Today, it is more difficult to update and improve the content of these medical examinations when the old medical protocols continue to be taken as a starting point when updates are discussed, resulting in only small amendments and without questioning their basis. A new basis is needed, the ‘*new why*’, when designing and performing mandatory medical examinations on workers.

To increase the chances of modernization in this field, medical professionals and scientists need to discuss the central topic, and that is why it is relevant and essential to change the content and process of mandatory medical examinations (pre-employment medicals and on-employment medicals). As a local example, Dutch legislation provides a contrasting view compared with many existing (inter)national medical protocols for specific occupations. Four issues are discussed which clarify how the Dutch view may differ from ideas in other countries. In the Netherlands, new ‘*why*’ principles for occupational physicians should guide the ‘*how*’ and ‘*what*’ in designing and performing periodic medical examinations. After amendments were made in Dutch law on pre-employment medicals, two guidance documents for medical professionals in occupational medicine were developed: one on pre-employment medical examinations [2] and one on on-employment medical examinations [3].

Firstly, in these guidance documents, it is stated that assessing a workers’ medical fitness for work should be based on the specific but current health and safety requirements that exist *in performing the job*, and not on general rules concerning the average impact of diseases, nor on questions about a workers’ general health. The employer plays a role in recognizing the specific job demands and has to check whether risks due to these job demands could be prevented in order to avoid workers being medically screened unnecessarily. When no improvements are possible, the associated health or safety requirements needed for a specific job should be further described (by the occupational physician). These should include mental, physical, sensory, emotional and biomechanical requirements and be defined with as much job-specific detail as possible. This information forms the basis on which to select the most appropriate tests during the medical examination. Most safety requirements in a job such as a train driver, for example, deal with job demands that require specific aspects of vigilance and clear judgement [4]. Translating these requirements into (medical) tests during a medical examination should lead to different choices than the ones we encounter in more traditional medical examinations.

Secondly, and in line with the first point, we assume that the assessment of health complaints or diseases alone cannot be sufficient to detect workers with possible and relevant work limitations, given the precise requirements. We need specific signal questions in which certain relevant health complaints are coupled to decreased ability given the defined requirements needed for performing the job demands. This is why job-specific medical examinations with more precise questions included in the examination protocol with direct association to the defined requirements were developed.

Thirdly, the ‘standard’ solution in medical examination protocols of including clinical tests normally used to build a diagnostic framework (i.e. blood testing, urine testing, X-rays, etc.) is questionable and might not be the instruments needed in the preventive medical setting. Neither is it logical either because with mandatory medicals it is the physician who invites a worker for an exam ination while in the classic medical setting the patient will visit the doctor or hospital due to health problems.

Fourthly, we assume that the better the medical exam ination is tailored to the job demands and health and safety requirements, the more likely it is that an occupational physician can take valid decisions and provide tailored advice to keep workers in the job and fit for work: this should be seen as the ultimate goal, while most classical medical protocols might end with the decision that an individual worker is ‘unfit for the job’. Here is where the ‘*new why*’ is obvious: the assessment of the medical fitness for work is not a goal in itself, because it should be maintained by timely interventions to remove health or safety issues, improve work limitations or sustain work ability. The interventions are also standardized, as are the tests and test outcomes.

This way of thinking is not typically Dutch. In research, several authors argue along the same lines when researching classical examples of jobs with high physical demands for which mandatory medical examinations have been redesigned, such as for firefighters and policemen. Jamnik *et al*. [5] published work to explain how in Canada legally defensible employment standards for prominent physically demanding public safety occupations could be developed and practised. Tipton *et al*. [6] expanded the processes involved in establishing minimum occupational fitness standards, highlighting the interplay that occurs between the choice of measurements and the decision that follows.

Opponents of the Dutch approach might argue that the classical clinical way of thinking is needed when workers have a specific medical history or a newly developed disease but this is no different from considering the ongoing abilities of workers in jobs with safety or health requirements. Also, the emphasis should be on knowledge of the job-specific health or safety requirements when examining sick workers. For example, when a new serious health issue like a stroke emerges in a professional driver, a doctor has to decide about that person’s fitness-to-drive.

Two recent scientific studies on the content of med ical examinations for a licence to drive a motorized vehicle have been published. They serve as an example of how the modernization of medical protocols can be hampered or enhanced. As an example of hampering modernization, Rapoport *et al*., an international group of physicians or researchers systematically evaluated the quality of nine national guidelines about driving with medical illnesses [7]. Although all guidelines were given low AGREE-II ratings on rigour of development, ap plicability and documentation, in neither the introduction, nor the discussion or conclusion were ‘*the why*’ or ‘*the what*’ of these guidelines discussed or questioned. An example of enhancing modernization is the paper by Ranchet *et al*. who used the additional evidence of selecting only tests that were close to the specific job demands [8]. They compared using classical medical findings only to using information that was closer to the actual requirements in the form of a practical fitness-to-drive test; their results revealed that physicians using only the medical recommendations were less likely to reject those people who posed an actual risk on the road.

Examples of job-specific medicals developed with the ‘*new why*’, ‘*how*’ and ‘*what*’ in the last decade have led to the modernization of mandatory medicals in our department for ambulance workers [9], firefighters [10], hospital physicians [11], rail safety workers [12] and workers in the construction industry [13]. This revised way of performing medical examinations has had a different impact on the practice of occupational physicians. The ambulance sector introduced a national registration system of medical examination outcomes in 2011 but progress in practice has only been present in the firefighting sector since 2015. There has been no practical impact in the construction industry, where evidence and content was created for 103 occupations [13]. Most recently, for the newly merged National Police Force in the Netherlands (>60,000 workers), our research provided the evidence to construct the content of periodical mandatory medical examinations for 37 specific jobs within the organization (J. S. Boschman *et al*., in preparation).

It is not an easy job to change occupational physicians’ way of thinking in practice and implement better medical examinations in the short term. One of the main reasons might be found in the basic training of physicians that is almost completely directed towards the medical setting in hospitals. This medical training is less relevant for their future work in public and occupational medicine. Occupational physicians must understand how their medical skills were formed during basic training and what they need to apply and show in the preventive occupational medicine context. It will be worth all the efforts to change their practice because it is all about guiding workers better from an occupational health perspective. These workers chose to be active in their specific jobs including the specific physical, mental and safety demands. When the ‘*new why*’ of mandatory medical examination is taken more seriously by all medical professionals, it will modernize the daily practice of occupational medicine when conducting mandatory medical examinations.
